# Sex & gender considerations in concussion research

**DOI:** 10.2217/cnc-2017-0015

**Published:** 2018-01-18

**Authors:** Tatyana Mollayeva, Graziella El-Khechen-Richandi, Angela Colantonio

**Affiliations:** 1 Acquired Brain Injury Lab, University of Toronto, Toronto, ON, M5G 1V7, Canada; 2 Rehabilitation Sciences Institute, University of Toronto, Toronto, ON, M5G 1V7, Canada; 3 Toronto Rehabilitation Institute, University Health Network, Toronto, ON, M5G 2A2, Canada

**Keywords:** assault, concussion, epidemiology, sex/gender, sport/work related, traumatic brain injury

## Abstract

The study of concussion, a common form of mild traumatic brain injury, has received increased notice over the last decade. Recently, more researchers have been addressing the historic paucity of attention over sex and gender influences on recovery outcomes after concussion. This development has led to exciting progress in our understanding of concussion incidence and outcomes. In this review, we will report on new findings from varying studies on sex differences in the epidemiology of concussion and clinical manifestations of mild traumatic brain injury/concussion, further discussing some key issues related to the integration of sex and gender in concussion research in a broad range of contexts, with recommendations to guide future research, along with sex- and gender-sensitive policy considerations.

Policy statements have been made by major north-American funding agencies regarding the requirements for the consideration of sex and gender in research across all phases of the research process as it is a fundamental practice for good science [1–4]. The WHO Disability and Rehabilitation Action Plan 2014–2021 also calls for age and sex disaggregated data in policy documents [5]. ‘Sex’ refers to a set of biological attributes in humans and animals that are dependent on chromosomes, hormone levels and reproductive anatomy [2,5]. ‘Gender’ on the other hand, refers to behavioral expressions, socially constructed roles, expressions and identities, which intersect with sex and other determinants of health [2,5], and can create varying postconcussive experiences and outcomes. Studying the complex and interconnected role of sex and gender in mild traumatic brain injury (mTBI)/concussion is not an easy undertaking. In embracing an unbiased comparison across genders (i.e., men and women), recognizing the biological (sex) differences between males and females, and acknowledging the complex and dynamic relationship that exists between sex, gender and other factors (income, education, social environments, health practices, etc.) [6], great strides have been made. To address the current understanding of mechanisms subserving the influence of sex on epidemiology of concussive sport and work-related injury and injury outcomes (i.e., sex-specific vulnerability), this review is focused on: the sex-specific epidemiology of concussion and sex-/gender-specific clinical manifestations in children and adults with concussion. It is conceivable that sleep dysfunction could potentially reflect on injury epidemiology and outcomes, and as such, we present some of the recent evidence on sleep changes in persons who sustained work-related concussion, in particular findings derived from our sex-sensitive sleep research program. Finally, in light of the great deal of evidence from recent studies converging on the conclusion that sex and gender affects injury involvement and outcomes, we will discuss the implications for future research. In this review we will follow the methodology of original studies and refer to females and males when research questions investigated outcomes through a set of biological attributes, while using women and men when outcomes concerned differences are through a sociocultural process, associated with behavior, stress and coping. We acknowledge, however, that sex and gender constructs are interconnected, not binary and isolating the influences of sex and gender in research results is not simple.

## Sex-specific epidemiology of concussion

In order to put mTBI/concussion into the public health significance perspective, the broader picture of TBI across all severity is important. Every year, TBI is a leading cause of death and disability worldwide with research consistently indicating that the youngest and the oldest members of the population are at greatest risk [7–10]. Historically, males have dominated the majority of TBI cases across the age continuum; with age-adjusted TBI-related hospitalization rates for males shown to be consistently higher than that of females throughout a number of decades [8,9]. Such differences in sex-specific rates were attributed to higher incidences of general injury among younger males, a variance between males and females in traditional societal roles and activities, as well as risk-taking behaviors [11,12], highlighting a gendered side of this epidemiology trend in TBI.

The majority of TBIs (i.e., 70–90%) fall under a ‘mild’ severity rating, based on acute injury characteristics criteria, with the rates of people seeking medical attention in the emergency department (ED) ranging from 569.4 in 2006 to 807.9 per 100,000 in 2012 [13]; these rates are considered an underestimation of actual occurrences as not all people seek medical attention after injury. Consequently, those who do not seek medical attention are captured in neither hospital-based studies on mTBI incidence, nor studies on recovery and/or outcome. Based on concerns that the true incidence of mTBI is unknown, the CDC labeled mTBI a ‘silent epidemic’ and have published several reports in the past decade to elevate public health awareness on mTBI impact [14].

### Sport-related/pediatric concussions: sex & gender influences

It has been largely accepted in the concussion literature and in consensus conferences that sex and gender may be risk factors in injury involvement or influence injury severity [15]. Many researchers working on the topic propose that females sustain concussions more often than males, have more neurologic deficits, a different symptom constellation and delayed resolution of symptoms [16–21]. These differences were proposed to be driven, for instance, by disparities in the neck musculature and head/neck stability in girls/women making them more susceptible to injury. It has also been proposed that females have lower biomechanical thresholds, increasing concussion susceptibility especially in female athletes. Hormonal factors such as circulating estrogens may also elicit a differential pain response in females compared with males [16–21]. Recently, menstrual cycle phase has been shown to impact recovery outcomes in females due to changes in hormonal level, specifically in the reduction of progesterone concentration after mTBI, which subsequently leads to a withdrawal from its proposed neuroprotective properties [22]. Furthermore, it has been identified that females report more concussion symptoms prior to the injury, which can persist and increase risk of poorer outcome [23]. Therefore, more persistent symptoms in girls and women may be affected by this pre-existing profile.

A recent study in the province of Ontario in Canada on concussion-related ED or physician visits by children and adolescents (i.e., aged 5–18), revealed a 5.5-fold increase in concussion rates from 2003 to 2013, driven mainly by adolescents [24]. Although the total number of visits were higher for males than females across age groups, the overall increase in rates was greatest in females, with a 6.3-fold increase for females compared with a 3.6-fold increase in males, respectively. It remains unclear whether the difference in seeking medical attention is higher in females after injury due to disparities in symptom load, reactivity, behaviors or gender influences. It is hypothesized that girls/women may report more symptoms as it is more socially acceptable for women to admit vulnerability than for men [25]. At the same time, research reported that girls/women who display more traditional ‘masculine’ traits are more prone to risk-taking behaviors and may continue playing despite concussive symptoms [25] Other gender-related factors that may affect differential rates in seeking medical care are the quality of coaching and support for concussion care that may not be at the same level of that of boys/men.

A study of school-aged children and youth (3–18 years of age) with a diagnosis of concussion in Ontario has also revealed an increase in the reporting rates of concussions by sex between 2003 and 2011. Although in 2003, there were 8736 concussions treated in either an ED or a physician office, the number increased to 14,886 in 2010, with the rates for boys being 467 and 754, and for girls 209 and 441 per 100,000 Ontarians in 2004 and 2011, respectively [26]. Falls were also found to be the most common cause of concussion regardless of sex, accounting for 34% of all ED visits, followed by exposure to force (26%) and motor vehicle collisions (12.3%). For both boys and girls, the number of visits for concussion steadily increased until 15 years of age, then decreased until 18 years of age. Upon examining circumstances of injury, hockey/skating were the most common cause among school-aged children and youth [26].

Sport and recreation remain a major cause of mTBI in adolescents and young adults (17–20 years). Updates on sex-specific epidemiologic trends for sport-related concussion rates have been recently provided by Covassin *et al*. in studying athletes of the National Collegiate Athletic Association. Researchers demonstrated that females participating in sex-comparable sports (i.e., basketball, soccer) had a 1.4 times greater incidence of reported concussion than males, with some variability in reported incidence observed between sport types. Specifically, female softball players presented with almost  a twofold higher incidence of concussion compared with female baseball players. Female soccer and basketball players had a 1.5-times greater incidence of a reported concussion than their male counterparts involved in the same sport activities [27]. It is important to note, that all rates were calculated based on self-reported measures, which may represent an inherently different overall reporting on injury between males and females.

### Older Adults

Although the athletic population and youth specifically tend to dominate the concussion literature, the CDC recently released a report displaying significant rise in the incidence of ED cases of TBI among older adults [28]. Rates of ED visits for mTBI were 386 per 100,000 for persons aged 65–74 years, 777 per 100,000 persons aged 75–84 years and 1205 per 100,000 among those older than 84 years. Importantly, rates for females were higher than that for males, with 706 per 100,000 and 516 per 100,000, respectively. Ryu *et al*. were the first to provide estimates of mTBI incidences in the adult population of Ontario [29]. They examined cases of patients from a number of family physician offices and EDs across five different geographical regions in Ontario. The incidence rates in the ED were in the range of 426–535 per 100,000 Ontarians, and with added family physicians visits, the rates ranged between 493-653 per 100,000, with males accounting for 56% of all visits [29]. These findings are of considerable importance, as advanced age has been associated with poorer outcomes after TBI in general (i.e., greater risk of dying in the hospital), and also require greater healthcare assistance after discharge.

### Work-related concussions

Although sport-related concussions have dominated the discussion on concussions, a new awareness of the impact of concussion at the civilian workplace has recently emerged. According to data by the US Bureau of Labor Statistics (2016), the number of nonfatal occupational head injuries in 2015 totaled 94,360 in private, state and local government with an incidence rate of 8.5 per 10,000 [30]. The number of head injuries in private industries totaled to 74,180 with an incidence rate of 7.7 per 10,000. Due to the median number of 3 days away from work, these injuries are assumed to be of mild severity. The context of TBI in the workplace is also highly gendered. Serious and fatal injuries occur predominantly among males, however, when all levels of severity are included, women make up more than 40% of injuries [31]. High rates of injury were among government workers, specifically those in education and healthcare, which were predominately women [31]. These statistics indicate that a more tailored approach by sex and gender is needed in relation to a workplace context in terms of prevention.

Recently, the Ontario Workplace Safety and Insurance Board's (2016) Statistical Report showed that injuries coded as ‘concussions’ have increased from 0.6% in 2002 to 5% in 2015, indicating a 800% increase [32]. Data on cranial region injuries that were mostly concussions, showed that younger males in the 20–24 years of age group and older females in the 50–54 age-group for specific occupations were most affected. [32].

To support preventive efforts in mTBI/concussion-related occupational health and safety, our group carried out a series of studies in Canada's largest adult rehabilitation teaching hospital that evaluates injured workers with persistent symptoms after brain injury [33–37]. Consistent with results of a recent systematic review of 24 studies, it was found that male workers especially those in the youngest and oldest age groups, working in the primary (e.g., agriculture, forestry, mining) or construction industries were more likely to sustain a work-related TBI (wrTBI), with falls being the most common mechanism of injury regardless of injury severity [38]. Our unique worker-specific data (i.e., wrTBI, mostly concussions) emphasize that male workers were significantly more likely than female workers to be employed in the skilled agricultural and forestry occupational sectors, including assemblers and plant machine operators at the time of injury, while female workers were more frequently occupied in the managerial, professional and associate professional-type roles, as well as clerical support and service and sales-related positions [37]. Moreover, differences between male and female workers were observed within the occupational hazard themes: male workers were more likely than female workers to experience a wrTBI as a result of a breakdown or a malfunction in the operation of equipment at the time of injury, while the hazard of unexplained human factor at the time of injury did not differ between sexes [37].

## Sex-specific trends in postinjury sleep & recovery after work-related mTBI/concussions

When investigating common complaints in workers who experienced delayed recovery from mTBI/concussion, we found that two-thirds of the sample suffered from clinical insomnia [35]. The severity of insomnia was found to be a main factor independently associated with community integration [34], perceived disability severity [33], more severe fatigue and diminished alertness [36]. Consistent with previous research [39], we observed a trend toward better community integration in females compared with males, with scores 15.56 ± 6.01 versus 13.96 ± 4.67, respectively, when utilizing the community integration questionnaire, although our results did not reach the level of statistical significance [35]. Likewise, the frequency of marked/extreme global disability in females was lower than in males (36 vs 65%, respectively) when utilizing the Sheehan disability scale [34]. These results which focus on functional outcomes versus severity may challenge the commonly held view of poorer outcomes for females compared with males, in work-related concussions, regardless of the injury mechanisms. However, scales such as the community integration scale have been found to reflect higher scores in women because of overall greater participation in home integration reflecting gendered norms [40].

When we studied postconcussive symptoms of fatigue, alertness and daytime sleepiness through a sex lens, females did indeed report more severe fatigue than males (48.8 ± 12.8 vs 43.5 ± 15.9, as measured by the Fatigue Severity Scale), lower alertness (19.1 ± 9.8 vs 21.3 ± 9.2 as measured by the Toronto Hospital Alertness Test) and slightly higher daytime sleepiness (8.9 ± 5.7 vs 8.5 ± 5.7, as measured by the Epworth Sleepiness Scale), although neither of these results have reached the level of statistical significance in a bivariate analysis (p = 0.09, p = 0.269, p = 0.770, respectively) [41]. In fully adjusted linear regression models (i.e., for all known factors associated with these states), only alertness appeared to be sex-dependent, with males remaining more alert compared with females, however, male sex accounted for just slightly over 3% of variance in alertness [41].

Earlier research consistently reports sleep deprivation and falling asleep at the wheel due to shift work as reasons for many fatal crashes and traffic accidents [42,43]. We expanded on these studies and demonstrated that in a sample of persons experiencing delayed recovery from mTBI/concussion, almost half (49% of the sample) were working shifts at the time of injury, with sex differences observed in the type of shift work: females more often performed fast-rotating shifts, while males performed slow-rotating shifts [39]. Past research has been consistent with regard to the impact of shift work: a study of nurses reported that 79.5% of those working the night shift reported at least one drowsy driving incident, equal to an increased odds ratio of 3.96 (95% CI: 3.24–4.84) relative to nurses working the day shift; another study also linked shift work to elevated risk of workplace incidents, at 9%, and found the risk to be more pronounced for women [44–46].

Using a qualitative approach, we have also identified gender influences concerning return to work among men and women with mTBI. We found that the breadwinner role is important to both men and women, with support of coworkers also found to be important for both. However, more support was found in more traditionally ‘feminine’ environments versus more traditionally ‘masculine’ occupational contexts, with women more likely to seek out assistance [47]. Specific work-related concussion knowledge transfer resources can be found on the following website [48].

Sex as a risk factor for specific primary sleep disorders in mTBI/concussions has gained significant recent attention in the clinical and research communities. In our study, we found that while sleep-related variables in general explain the majority of variance in chronic pain in both males and females of middle age with concussion, the exact variables of great importance were sex-specific [49]. Thus, in males with concussions it was insomnia that alone explained 25% of pain severity, while in females, sleep-disordered breathing and excessive daytime sleepiness accounted for more than 24% of variance in pain severity in a model adjusted for socio-demographic, injury-related, occupational and psychosocial factors simultaneously [49]. These results are noteworthy and reinforce findings of previous research reporting that obstructive sleep apnea, a form of sleep-related breathing disorder (SRBD), remains largely undiagnosed in females, due to variations in clinical presentation (i.e., SRBD in females manifests with symptoms of depressive mood, morning headaches vs snoring and witnessed apnea in males, which is more diagnostically apparent) as well as higher tolerance to symptoms [50]. At the same time, sleep architecture is as severely affected by SRBD in females as in males [51]. Further efforts should be directed toward description of primary sleep disorders and their sex-specific manifestations and burden in mTBI/concussion. Since women are more likely to be responsible for childcare and household work, in an effort to balance work and home life, in addition to primary sleep disorders they might be cutting back on sleep duration placing themselves in additional risk; cutting back on sleep is a common practice for 70% of night-time workers and 63% of rotating-shift workers, regardless of sex, according to 2008 Statistics Canada [52]. Overall, occupational brain injury in the civilian population requires a tailored focus to address specific needs.

## Intimate partner violence, workplace assaults & concussions

Studies on concussions in the context of intimate partner violence (IPV) and workplace assault have been sparse. IPV differentially affects women, which can result in physical as well as psychological harm, in addition to involvement with the justice system [53]. Global estimates by WHO reveal that approximately one in three women who have been in a relationship report experiencing some form of physical and/or sexual violence by an intimate partner during their lifetime [54]. Common injuries of IPV survivors involve assaults inflicted to the face, head and neck, and/or resulting from strangulation [55], putting women at risk for brain injury. Elevated TBI rates of up to 80% in women survivors of IPV have been identified [56]. More research is needed on concussions in this vulnerable population. Likewise, when we studied assault-precipitated TBIs in Ontario, which were largely comprised of concussions/mild injury [57], sex appeared implicated in several patterns of victimization: females were at greater risk for sustaining the injury than male workers (59.1 vs 40.9%), in addition to injuries to the head and neck, females sustained more injuries to other body parts compared with males. Females also had higher rates of injury in the healthcare/social services and educational occupational sectors, while male workers showed the same pattern in the law enforcement/public administration sector [58].

## Critical perspective

Our review provides compelling evidence that rates and prognosis of concussion are subject to both gender (i.e., the result of a socio-cultural process, associated with risk taking behavior, stress and coping) and sex (i.e., a set of biological attributes such as concentration of sexual hormones, different expression of genes on X and Y chromosomes, etc., associated with symptoms severity and outcomes), and highlights the feasibility and necessity of conducting epidemiological, clinical and patient-centered research simultaneously, to address the very real contextual challenges of sex and gender in concussive injury, which is critical for the prevention and management of this injury at the population level, and among vulnerable populations.

## Knowledge transfer

In February of 2016, an entire special issue was published in the Archives of Physical Medicine and Rehabilitation entitled Sex, Gender and Traumatic Brain Injury, which included articles on mTBI [58]. One of the articles focused on a re-analysis of a systematic review on prognosis after brain injury [59]. The study found that among the 200 studies of prognosis after mTBI, only 7% of studies provided sex-stratified data. Although overall studies in the literature may model sex or include the sex distribution, there is a huge paucity of sex-stratified data in the field of concussion [59]. Systematic reviews in general do not take an explicit focus on sex and gender when synthesizing results. The authors have also advocated for systematic review tools that consider sex and gender to be utilized. This lack of sex-specific data, however, also affects informing guidelines for practice. To our knowledge, there are no concussion guidelines that provide explicit considerations for sex by age. For instance, the Ontario Neurotrauma Foundation Concussion Guidelines currently have no references to sex and gender to date [60]. New concussion guidelines from a recent conference held in Berlin have not made explicit considerations on this topic [61].

The senior author is the founding chair and now co-chair of an International Task Force on Girls and Women with Acquired Brain Injury in collaboration with the American Congress of Rehabilitation Medicine. The task force was an outcome of the first international workshop on girls and women with TBI, led by the senior author, which was held in Montreal in 2010 and funded by the Canadian Institutes for Health Research (CIHR) in collaboration with the American Congress of Rehabilitation Medicine [62]. This is a network of scientists, clinicians and persons with lived experiences of brain injury and those that represent them [63]. This work through the task force involves creating scholarship such as through a special issue, conducting systematic reviews with a sex and gender lens, planning a symposium that addresses women and TBI, as well as advocacy for journal editors to adapt reporting that addresses sex and gender. The task force has broadened its scope from a more exclusive focus on girls and women to include the influence of sex and gender overall. There are also a growing number of online resources for scientists and trainees that provide substantive and methodological support for the integration of sex and gender in research. These include the CIHR Institute for Gender and Health website and the NIH website.

## Future perspective

Although there is no question that numerous injury, health and recovery indicators among persons with concussions are sex- and gender-specific, there is still a great deal of knowledge about sex, gender and concussion needed, for results to make their way into policy and practice. The current research is dominated by sports-related concussion [64]. It is not clear to what extent this research adequately informs both prevention and management regarding concussion, in other contexts, such as the workplace. Further, more attention is needed with respect to research on older adults, a growing segment of the population. Currently, concussion guidelines, to our knowledge, have not made any recommendations on how to manage sex and gender in clinical settings, which warrants explicit consideration. To ensure greater precision in concussion-related policy and practice, integration of sex and gender diversity into all associated activity is greatly needed. This can be done by investigating how the sex of injured persons relate to their social location/societal position and access to healthcare resources; whether male and female persons hold different lifestyle, occupational and family responsibilities before and after the injury, and make different behavioral choices and attitudes toward activities associated with greater risk of concussion; and reflecting these attitudes and behaviors through the lens of relevant gender relations and sex-linked biology. It is anticipated that research advances in these areas will culminate in the development of tailored policy interventions that will be applied to prevent injuries and improve outcomes of all injured persons, regardless of their gender or sex.

**Executive summary**

- The theoretical and clinical constructs of sex and gender are inevitably overlapping, with social roles driving or modifying variances between men or women in traumatic brain injury (TBI) rates, recovery course and outcomes.
- Epidemiological data consistently indicate that the youngest and oldest members of the population are at greatest risk of mild TBI; the data also reveal higher rates of concussions for females compared with males in sex-comparable sports; the reason for this trend is not completely understood.
- Although the clinical data support higher symptom loads long term after work-related concussions in females when compared with males, their community integration and perceived disability were less adversely affected; in both sexes, sleep functioning was observed to be associated with various recovery outcomes.
- Gender–sex interactions determine rates and severity of TBI, help-seeking behaviors and healthcare system use, and therefore should be reflected in policy and practice.

## References

[1] Health Canada Sex and gender-based analysis (SGBA). http://www.hc-sc.gc.ca/hl-vs/gender-genre/analys/gender-sexes-eng.php.

[2] Clayton JA, Tannenbaum C (2017). Sex and gender reporting in research–reply. *JAMA*.

[3] Tannenbaum C, Greaves L, Graham ID (2016). Why sex and gender matter in implementation research. *BMC Med Res Methodol.*.

[4] Legato MJ, Johnson PA, Manson JE (2016). Consideration of sex differences in medicine to improve health care and patient outcomes. *JAMA*.

[5] WHO The WHO Global Disability Action Plan 2014–2021. http://www.who.int/disabilities/actionplan/en/.

[6] Mollayeva T, Colantonio A (2017). Gender, sex and traumatic brain injury: transformative science to optimize patient outcomes. *Healthc. Q*.

[7] Leo P, McCrea M (2016). Translational research in traumatic brain injury. *Epidemiology*.

[8] Langlois JA, Kegler SR, Butler JA (2003). Traumatic brain injury-related hospital discharges. Results from a 14-state surveillance system, 1997. *MMWR Surveill. Summ.*.

[9] Taylor CA, Bell JM, Breiding MJ, Xu L (2017). Traumatic brain injury-related emergency department visits, hospitalizations, and deaths – United States, 2007 and 2013. *MMWR Surveill. Summ.*.

[10] Colantonio A, Saverino C, Zagorski B (2010). Hospitalizations and emergency department visits for TBI in Ontario. *Can. J. Neurol. Sci.*.

[11] Ilie G, Mann RE, Hamilton H (2015). Substance use and related harms among adolescents with and without traumatic brain injury. *J. Head Trauma Rehabil.*.

[12] Love PF, Tepas JJ, Wludyka PS, Masnita-Iusan C (2009). Fall-related pediatric brain injuries: the role of race, age, and sex. *J. Trauma*.

[13] Cancelliere C, Coronado VG, Taylor CA, Xu L (2017). Epidemiology of isolated versus nonisolated mild traumatic brain injury treated in emergency departments in the United States, 2006–2012: sociodemographic characteristics. *J. Head Trauma Rehabil.*.

[14] Langlois JA, Marr A, Mitchko J, Johnson RL (2005). Tracking the silent epidemic and educating the public: CDC's traumatic brain injury-associated activities under the TBI Act of 1996 and the Children's Health Act of 2000. *J. Head Trauma Rehabil.*.

[15] McCrory P, Meeuwisse WH, Aubry M (2013). Consensus statement on concussion in sport: the 4th International Conference on Concussion in Sport held in Zurich, November 2012. *J. Am. Coll. Surg.*.

[16] Covassin T, Elbin R, Kontos A (2010). Investigating baseline neurocognitive performance between male and female athletes with a history of multiple concussion. *J. Neurol. Neurosurg. Psychiatry*.

[17] Covassin T, Moran R, Elbin RJ (2016). Sex differences in reported concussion injury rates and time loss from participation: an update of the National Collegiate Athletic Association Injury Surveillance Program from 2004–2005 through 2008–2009. *J. Athl. Train.*.

[18] Covassin T, Schatz P, Swanik CB (2007). Sex differences in neuropsychological function and post-concussion symptoms of concussed collegiate athletes. *Neurosurgery*.

[19] Covassin T, Swanik CB, Sachs ML (2003). Sex differences and the incidence of concussions among collegiate athletes. *J. Athl. Train.*.

[20] Covassin T, Swanik CB, Sachs M (2006). Sex differences in baseline neuropsychological function and concussion symptoms of collegiate athletes. *Br. J. Sports Med.*.

[21] Broshek DK, Kaushik T, Freeman JR (2005). Sex differences in outcome following sports-related concussion. *J. Neurosurg.*.

[22] Wunderle K, Hoeger KM, Wasserman E, Bazarian JJ (2013). Menstrual phase as predictor of outcome after mild traumatic brain injury in women. *J. Head Trauma Rehabil.*.

[23] Brown DA, Elsass JA, Miller AJ (2015). Differences in symptom reporting between males and females at baseline and after a sports-related concussion: a systematic review and meta-analysis. *Sports Med.*.

[24] Zemek LR, Grool MA, Duque RD (2017). Annual and seasonal trends in ambulatory visits for pediatric concussion in Ontario between 2003 and 2013. *J. Pediatr.*.

[25] Kroshus E, Baugh CM, Stein CJ, Austin SB, Calzo JP (2017). Concussion reporting, sex, and conformity to traditional gender norms in young adults. *J. Adolesc.*.

[26] Macpherson A, Fridman L, Scolnik M, Corallo A, Guttmann A (2014). A population-based study of paediatric emergency department and office visits for concussions from 2003 to 2010. *Paediatr. Child Health*.

[27] Covassin T, Moran R, Elbin RJ (2016). Sex differences in reported concussion injury rates and time loss from participation: an update of the National Collegiate Athletic Association Injury Surveillance Program from 2004–2005 through 2008–2009. *J. Athl. Train.*.

[28] Albrecht JS, Hirshon MJ, McCunn M (2016). Increased rates of mild traumatic brain injury among older adults in US emergency departments, 2009–2010. *J. Head Trauma Rehabil.*.

[29] Ryu WHA, Feinstein A, Colantonio A, Streiner DL, Dawson DR (2009). Early identification and incidence of mild TBI in Ontario. *Can. J. Neurol Sci.*.

[30] U.S. Department of Labor Bureau of Labor Statistics. http://www.bls.gov/news.release/pdf/osh2.pdf.

[31] Colantonio A, Mroczek D, Patel J, Lewko J, Fergenbaum J, Brison R (2010). Examining occupational traumatic brain injury in Ontario. *Can. J. Public Health*.

[32] Workplace Safety and Insurance Board. http://www.wsib.on.ca/WSIBPortal/faces/WSIBDetailPage?cGUID=WSIB066165&rDef=WSIB_RD_ARTICLE.

[33] Mollayeva T, Pratt B, Cassidy JD, Shapiro CM, Colantonio A (2016). The relationship between insomnia and disability in persons with delayed recovery from mild traumatic brain injury. *Sleep Med.*.

[34] Mollayeva T, Shapiro CM, Mollayeva S, Cassidy JD, Colantonio A (2015). Modeling community integration with workers with mild traumatic brain injury. *BMC Neurology*.

[35] Mollayeva T, Mollayeva S, Shapiro CM, Cassidy JD, Colantonio A (2016). Insomnia in workers with delayed recovery from mild traumatic brain injury. *Sleep Med.*.

[36] Scherer M, Belben T, Colantonio A, Thurairajah P, Mollayeva T (2015). The relationship between sleep, depression, and traumatic brain injury: a study of Ontario workers with head trauma. *J Sleep Disor. Treat Care*.

[37] Amodio V, Bruch H, Mollayeva T, Colantonio A (2017). Using the narratives of Ontarians with a work-related traumatic brain injury to inform injury prevention: a mixed methods approach. *WORK*.

[38] Chang VC, Guerriero EN, Colantonio A (2015). Epidemiology of work-related traumatic brain injury: a systematic review. *Am J. Ind. Med.*.

[39] Andelic N, Arango-Lasprilla JC, Perrin PB, Sigurdardottir S, Lu J (2016). Modeling of community integration trajectories in the first five years after traumatic brain injury. *J. Neurotrauma*.

[40] Willer B, Rosenthal M, Kreutzer JS, Gordon WA, Rempel R (1993). Assessment of community integration following rehabilitation for traumatic brain injury. *J. Head Trauma Rehabil.*.

[41] Mollayeva T, Shapiro CM, Cassidy D, Mollayeva S, Colantonio A (2017). Assessment of mild traumatic brain injury/concussion-related fatigue, alertness, and daytime sleepiness: a diagnostic modeling study. *Neuropsychiatry (London)*.

[42] Gurubhagavatula I, Patil S, Meoli A (2015). Sleep and transportation safety awareness task force of the American Academy of Sleep Medicine. Sleep apnea evaluation of commercial motor vehicle operators. *J. Clin. Sleep Med.*.

[43] Waage S, Pallesen S, Moen BE (2014). Predictors of shift work disorder among nurses: a longitudinal study. *Sleep Med.*.

[44] Luyster FS, Strollo PJ, Zee PC, Walsh JK (2012). Boards of directors of the American Academy of Sleep Medicine and the Sleep Research Society. Sleep: a health imperative. *Sleep*.

[45] Scott LD, Hwang WT, Rogers AE, Nysse T, Dean GE, Dinges DF (2007). The relationship between nurse work schedules, sleep duration, and drowsy driving. *Sleep*.

[46] Wong IS, Smith PM, Ibrahim S, Mustard CA, Gignac MA (2016). Mediating pathways and gender differences between shift work and subjective cognitive function. *Occup. Environ. Med.*.

[47] Stergiou-Kita M, Mansfield E, Colantonio A (2014). Injured workers’ perspectives on how workplace accommodations are conceptualized and delivered following electrical injuries. *J. Occup. Rehabil.*.

[48] Acquired Brain Injury Research Lab http://www.abiresearch.utoronto.ca/research/workrelatedtbi/resources/.

[49] Mollayeva T, Cassidy JD, Shapiro CM, Mollayeva S, Colantonio A (2017). Mild traumatic brain injury/concussion-related pain in males and females: a diagnostic modelling study. *Medicine (Baltimore)*.

[50] Mihai V, Rusu G, Mihescu T (2010). Demographic, clinical and polysomnographic differences between men and women. *Pneumologia*.

[51] Mollayeva T, Colantonio A, Cassidy JD, Vernich L, Moineddin R, Shapiro CM (2017). Sleep stage distribution in persons with mild traumatic brain injury: a polysomnographic study according to American Academy of Sleep Medicine standards. *Sleep Med.*.

[52] Statistics Canada. http://www.whsc.on.ca/Files/Resources/Hazard-Resource-Lines/Shift-Work-WHSC-Resource-Line.aspx.

[53] Synder RL (2016). No visible bruises: domestic violence and traumatic brain injury. http://www.newyorker.com/news/news-desk/the-unseen-victims-of-traumatic-brain-injury-from-domestic-violence.

[54] WHO (2016). Fact sheet. Violence against women: intimate partner and sexual violence against women. http://www.who.int/mediacentre/factsheets/fs239/en/Accessed.

[55] Sheridan DJ, Nash KR (2007). Acute injury patterns of intimate partner violence victims. *Trauma Violence Abuse*.

[56] Kwako LE, Glass N, Campbell J, Melvin KC, Barr T, Gill JM (2011). Traumatic brain injury in intimate partner violence: a critical review of outcomes and mechanisms. *Trauma Violence Abuse*.

[57] Mollayeva T, Mollayeva S, Lewko J, Colantonio A (2016). Work-related traumatic brain injury due to assault. *WORK*.

[58] Colantonio A (2016). Sex, gender, and traumatic brain injury: a commentary. *Arch. Phys. Med. Rehabil.*.

[59] Cancelliere C, Donovan J, Cassidy JD (2016). Is sex an indicator of prognosis after mild traumatic brain injury: a systematic analysis of the findings of the World Health Organization Collaborating Centre Task Force on Mild Traumatic Brain Injury and the International Collaboration on Mild Traumatic Brain Injury Prognosis. *Arch. Phys. Med. Rehabil.*.

[60] Ontario Neurotrauma Foundation (2017). New standards to guide post-concussion care in Ontario. http://onf.org/.

[61] McCrory P, Meeuwisse W, Dvorak J (2017). Consensus statement on concussion in sport-the 5th international conference on concussion in sport held in Berlin, October 2016. *Br. J. Sports Med.*.

[62] Harris JE, Colantonio A, Bushnik T (2012). Advancing the health and quality-of-life of girls and women after traumatic brain injury: workshop summary and recommendations. *Brain Inj.*.

[63] American Congress of Rehabilitation Medicine. http://acrm.org/about/.

[64] Iverson GL, Gardner AJ, Terry DP (2017). Predictors of clinical recovery from concussion: a systematic review. *Br. J. Sports Med.*.

